# Removal Effect of Atrazine in Co-Solution with Bisphenol A or Humic Acid by Different Activated Carbons

**DOI:** 10.3390/ma11122558

**Published:** 2018-12-16

**Authors:** Zhansheng Wu, Xinhui Wei, Yongtao Xue, Xiufang He, Xia Yang

**Affiliations:** 1 School of Environmental and Chemical Engineering, Xi’an Polytechnic University, Xi’an 710048, China; yangxia0701@163.com; 2 School of Chemistry and Chemical Engineering, Shihezi University, Shihezi 832003, China; weixinhui6@126.com (X.W.); hexiufang2013@163.com (X.H.)

**Keywords:** activated carbon, adsorption, atrazine, co-solution, bisphenol A, humic acids, NaCl

## Abstract

Activated carbons (ACs) based on apricot shells (AS), wood (W), and walnut shells (WS) were applied to adsorb atrazine in co-solutions. To study the effect of Bisphenol A (BPA) on the adsorption behavior of atrazine, the adsorption performance of ACs for BPA in single solution was studied. The results demonstrated that the adsorption kinetics of BPA fitted the pseudo-second-order model, the adsorption isotherms of BPA followed the Langmuir model. Meanwhile, the adsorption kinetics of atrazine fitted the pseudo-second-order kinetics model and the isotherm was consistent with the Freundlich model both in single solution and co-solution. In addition, competitive adsorption was observed when atrazine coexisted with BPA or humic acid. For the adsorption capacity, the adsorption amount of ASAC, WAC, and WSAC for atrazine obviously decreased by 18.0%, 30.0%, and 30.3% in the presence of BPA, respectively, which was due to the π−π interactions, hydrophobic interactions, and H-bonds, resulting in the competitive adsorption between atrazine and BPA. This study contributes to the further understanding of the adsorption behavior for atrazine in co-solution.

## 1. Introduction

Atrazine (2-chloro-4-(ethylamino)-6-isopropylamino-striazine), a typical endocrine disrupting substance [1], has been widely used in agricultural production to efficiently control broad leaves and annual weeds, which canenhance the crop yields and farm efficiency [2]. Atrazine is durable and highly mobile, and pollutes surface water and groundwater, which may do great harms to human health by affecting the central nervous, endocrine and immune systems [3]. However, Atrazine is difficult to be removed from contaminated environmental media due to its easy mobility, long-term preservation, and natural stability [4].

According to the reports, adsorption isregarded as a promising and effective method in wastewater treatment [5], providing a fast treatment for the pollutants removal [6,7]. In particular, with readily availability and low cost of materials, the adsorption effect of activated carbons (ACs) on natural organic compounds, metal ions, and synthetic organic matter is very effective [8,9]. ACs can be easily obtained from different agricultural by-products such as nuts, wheat, straw, soybeans, corn stover, and wood [10,11,12,13]. Therefore, pollutants removal through the adsorption onto ACs is an effective method.

To date, numerous studies have proved that atrazine removal through the adsorption of ACs is an effective method, but the adsorption performances and behaviors of different ACs are significantly different. For example, atrazine removal using ACs from wheat straw is via forming the π bond with atrazine in the adsorption process [11]; atrazine removal using ACs from corn stalks, poultry, rice straws, and pig manures is influenced mainly by the functional groups, which has small surface areas [12,14]. When atrazine coexists with Cu (II) in co-solution, the Cu (II) complexes can shield the hydrophobic and hydrophilic sites (carboxyl and hydroxyl) of atrazine adsorption [15]. However, the atrazine wastewater usually contains a variety of pollutants in a nature environment, and few studies had reported the effect of different substances in co-solution on the adsorption behavior of atrazine onto different adsorbents. Therefore, it is of great significance to study the adsorption behavior of atrazine in co-solution. 

Bisphenol A (BPA) (2, 2-(4, 4-dihydroxy phenyl) propane), a typical endocrine disrupting substance [1], has been widely used as monomer for product food packaging, polyvinyl chloride (PVC), dental sealants, and baby bottles [16]. It can interfere with the endocrine system, and has hostile impacts on the environment even at trace level [17]. Humic acid (HA), from the decomposition of animal and plant matters in aquatic and terrestrial systems [18], is the most important active ingredient in soil, sediment, and surface water as well as the main natural organic matter (NOM) in groundwater. In addition, the NaCl, a non-negligible factor in the wastewater environment, which needs to be considered in the pollutant removal. In the natural environment, atrazine is likely to coexist with BPA, HA, and NaCl. However, the effect of BPA, HA, and NaCl on the adsorption of atrazine has received little attention. Thus, there are great application values to determine the effect of BPA, HA, and NaCl on the adsorption of atrazine. 

Hence, this study mainly focuses on researching the adsorption performance of atrazine on ASAC, WAC, and WSAC in co-solution. To determine the adsorption kinetics and isotherms of atrazine in co-solution with BPA, the adsorption kinetics and isotherms of BPA in a single solution were analyzed. The effect of adsorption performance of atrazine in the presence of BPA was also studied, and the mechanism of competitive adsorption of atrazine and bisphenol A was discussed.Besides, the effect of the HA and NaCl on adsorption behavior of atrazine onto ASAC, WAC, and WSAC were determined. 

## 2. Experiment

### 2.1. Materials and Methods

The WAC (wood), WSAC (walnut), and ASAC (apricot shells) were purchased from Henan Zhongbang Environmental Technology Co., Ltd. (Zhengzhou, China). The WAC, WSAC, and ASAC had been characterized by element analysis, BET analysis, and XPS analysis in our previous research (Table 1) [19].

The chemical reagents and chemicals used were analytical grade. Atrazine and BPA were purchased from Nanjing Zhongli New Material Technology Co., Ltd. (Nanjing, China) and Shanghai Maclean Biochemical Technology Co., Ltd. (Shanghai, China), respectively. HA and NaCl were purchased from Adamas. The major physicochemical properties of atrazine and BPA were shown in Table 2. 

### 2.2. Adsorption of Atrazine and BPA onto the AC Samples

#### 2.2.1. Adsorption of Atrazine Only 

Atrazine solution (50 mL) and ACs (20.0 mg) were added into a 100 mL conical flask, and then sealed and agitated at 160 rpm in a water bath shaker (SHZ-B, Bo Xun Industrial Co., Ltd, Shanghai, China). The adsorption kinetics of atrazine (90 mg/L) onto ACs were studied at different adsorption times (1 s to 12 h) at 35 °C. The adsorption isotherms of atrazine were determined at different initial concentrations (60, 70, 80, 90, and 100 mg/L). The residual concentrations of atrazine were detected withHigh Performance Liquid Chromatography (HPLC, Agilent Technologies 1010, PaloAlto, CA, USA) equipped with a reversed-phase C18 column (5 mm, 4.6 mm×150 mm) at a wavelength of 222 nm. The mobile phase was 30:70 (v:v) of ultrapure water and methanol. Deionized water was used in the whole experiment. The pH of the solution was not adjustedfurther and the initial pH was 7.05.

#### 2.2.2. Adsorption of BPA Only

The adsorption experiments were carried out with 50 mL BPA solution (80 mg/L) and 20.0 mg of adsorbents in a 100 mL conical flask. The method for adsorption kinetics determination was the same as the above description in 2.2.1. The adsorption isotherms of BPA were analyzed at different initial concentrations (20, 40, 60, 80, and 100 mg/L) at 35 °C. The residual concentrations of BPA were detected by HPLC at a wavelength of 278 nm. The mobile phase was 30:70 (v:v) of ultrapure water and methanol, at a flow of 1 mL/min.

#### 2.2.3. Adsorption of Atrazine inCo-Solution

Atrazine and BPA co-solution was used by dissolving in 5% (v/v) ethanol solution considering the low solubility of atrazine and BPA in water. The batch adsorption experiments were carried out with 50 mL of co-solution and 20.0 mg of ACs in a 100 mL conical flask, and then sealed and agitated at 160 rpm in a water bath shaker. The adsorption kinetics of atrazine (90 mg/L) with BPA (50 mg/L) in co-solution were evaluated at different adsorption times (1 s to 12 h) at 35 °C. The adsorption isotherms of atrazine were analyzed at different initial concentrations (60, 70, 80, 90, and 100 mg/L) of atrazine with BPA (50 mg/L) in co-solution at 35 °C.

The effect of HA on the adsorption of atrazine was determined in different initial concentrations (60, 70, 80, 90, and 100 mg/L) of atrazine with HA (20 mg/L) onto the ACs in co-solution at 35 °C for 12 h. The effect of NaCl on adsorption of atrazine was evaluated at the initial concentration (90 mg/L) of atrazine with different initial concentrations of NaCl onto the samples in co-solution at 35 °C for 12h. The residual concentration of atrazine was detected by HPLC. 

### 2.3. Data Calculation

The adsorption amount of the adsorbate (*q*_t_, mg/g) was calculated according to the following Equation (1):

*q*_t_ = (*C*_0_ − *C*_t_) *V*/*M*, (1)

where *C*_0_ and *C*_t_ (mg/L) are the concentrations at the initial and given time t, respectively. *V* (mL) is the volume of the adsorbate solution, and *M* (mg) is the mass of the AC added to the flask. 

The adsorption kinetics models, pseudo-first-order (Equation (2)), and pseudo-second-order (Equation (3)) were used to identify the adsorption equilibrium time [19].

*q*_t_ = *q*_e_ (1 − e^−*k*^_1_*^t^*), (2)

*t*/*q*_t_ = 1/(*k*_2_*q*_e_^2^) + *t*/*q*_e_, (3)

where *q*_e_ and *q*_t_ (mg/g) are the adsorption amounts for the adsorbates onto the AC at the equilibrium and any time t (h), respectively. *k*_1_ (h^−1^) and *k*_2_ (g/(mg∙h)) are the kinetic constants.

The intra-particle diffusion model (Equation (4)) and film diffusion model (Equation (5)) were used [21] to identify diffusion mechanism of adsorption.

*q*_t_ = *k*_p_·*t*^1/2^ + C, (4)

−ln(1 − *q*_t_/*q*_e_) = *k*_bf_*t*, (5)

where *k*_p_mg/(g∙h^1/2^) and *k*_bf_ (h^−1^) are rate constants for intra-particle diffusion and liquid film diffusion, respectively.

The experimental data were fitted with Langmuir (Equation (6)), Freundlich (Equation (7)), Temkin (Equation (8)), and Dubinin–Radushkevich (D–R) (Equation (9)) isotherm models [22]. The residual root-mean squared error (RMSE) [19] function values can be analyzed using the Equation (10):

*q*_e_ = *q*_m_*K*_L_*C*_e_/(1 + *K*_L_*C*_e_), (6)

*q*_e_ = *K*_F_*C*_e_^1/*n*^, (7)

*q*_e_ = *B ln* (*ACe*), (8)

*Lnq_e_* = *lnq_m_* − *K*_d_*ln*(*1* + *1*/*Ce*), (9)

*RMSE* = [∑(*q*_e exp n_ − *q*_e cal n_)^2^ /(n − 1)]^1/2^, (10)

*R*_L_ = 1/(1 + *C*_m_*K*_L_*)*, (11)

where *q*_m_ (mg/g) is the maximum adsorption capacity; *q*_e_ (mg/g) is the adsorption amount of the adsorbate onto the adsorbents at the equilibrium time; *K*_L_ (L/mg) and *K*_F_ (L/mg) are the Langmuir and Freundlich constants, respectively; *n* is the empirical parameter; *b* (J/mol), *A* (L/mg), R (8.314 J/(mol∙K)), and *T* (K) are the Temkin constants related to the thermal constant of adsorption, equilibrium binding constant, gas constant, and absolute temperature, respectively; *K*_d_ = R^2^*T*^2^/*E*^2^; *E* (J/mol) is the constant related to free energy; and *R*_L_ is the dimensionless separation factor.

## 3. Results and Discussion

Table 3 displays the result of Boehm titrationand pH_PZC_. ASAC samples had the highest number of oxygen-containing functional groups and the lowest pH_PZC_. In addition, the amount of carboxyl groups, lactone groups, and carbonyl groups in WSAC was also reduced, and the number of surface basic functional groups was increased compared with ASAC, which was consistent with the results of XPS. At the same time, the reduction of acidic groups on the surface of activated carbons and the increase of pH_PZC_ may be beneficial to the adsorption of pollutants.

### 3.1. Adsorption Kinetics

#### 3.1.1. Adsorption Kineticsof BPA onto ACs 

**Figure 1:**
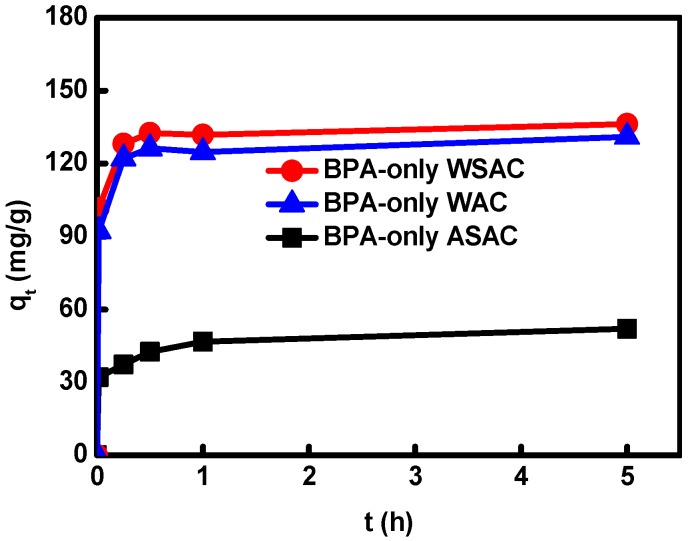
The adsorption kinetic curves of BPA onto ACs in single solution.

The adsorption behaviors of BPA onto WAC, WSAC, and ASAC were similar. The adsorption rates of BPA on WAC, WSAC, and ASAC were the fastest during the first 15 min, thereafter the adsorption rates slowed down, which was caused by the reduced amount of active sites of the adsorbents with the reaction going on [23]. When the adsorption progress reached equilibrium at 5 h, the order of adsorption capacity for BPA followed WSAC > WAC > ASAC (Figure 1). It is mainly determined by the order of BET surface areas, pore volume, and micropore volume: WSAC > WAC > ASAC (Table 1), because the pore structure and large surface area of ACs could increase the adsorption capacity for BPA in aqueous solution. What’s more, the polarity order of ACs was: WAC < WSAC < ASAC, and their hydrophobicity order was: WSAC > WAC > ASAC (please refer in the reference 19, hydrophobicity and aromaticity were also characterized in that paper). The lower polarity and higher hydrophobicity of WSAC was conducive to remove BPA by hydrophobic interactions. In addition, compared with WSAC and ASAC, the high aromaticity appearing in WAC could lead to the forming of the π–π interactions between aromatic ring of BPA and the aromatic compounds in samples to remove BPA from solution. 

Furthermore, ACs might adsorb BPA by H-bonding interactions through the hydrophilic oxygen-containing groups of ACs reacting with hydroxyl groups of BPA [20]. While ASAC had more oxygen-containing groups than WSAC and WAC, leading to the lower adsorption capacity, which implied that H-bonding interactions were not the main factor that affected the adsorption of BPA, and the acidic oxygen-containing group of samples was not conducive to the removal of BPA [20]. Since the water molecule clusters formed by the acidic oxygen-containing groups on the samples adsorbed more water molecules from the solution, it prevented the target organic substances from entering the hydrophobic area of the ACs, and the acidic oxygen-containing group of samples weakened the sample’s ability to serve as a π donor, affecting the π−π dispersion interaction between the BPA aromatic ring and the aromatic compounds in sample [20,24]. In addition, the adsorption capacity of WAC (the lowest in acidic oxygen-containing groups) for BPA is higher compared with that of ASAC, which indicated that the adsorption mechanism of BPA onto WAC might be mainly affected by π–π interactions instead of H-bonding interaction [20]. Thus, the adsorption amount of WSAC for BPA was higher than that of WAC, indicating that the effect of surface area and pore structure on the adsorption of BPA was higher than that of chemical characters. The adsorption mechanism of WSAC for BPA was not only affected by π–π interactions and hydrophobic interactions, but also affected by H-bonding interactions [20]. Moreover, electrostatic attraction/repulsion between BPA and samples would occur becauseof surface charges. The adsorption kinetic parameters of BPA onto sample displayed better accordance with the pseudo-second-order model than pseudo-first-order model in Table 4. The *q*_e,cal_ from pseudo-second-order model was consistent with the experimental data well. Therefore, the adsorption process of BPA onto WAC, WSAC, and ASAC can be well described by the pseudo-second-order kinetics model.

#### 3.1.2. Influence of BPA on Adsorption Kinetics of Atrazine onto ACs.

**Figure 2:**
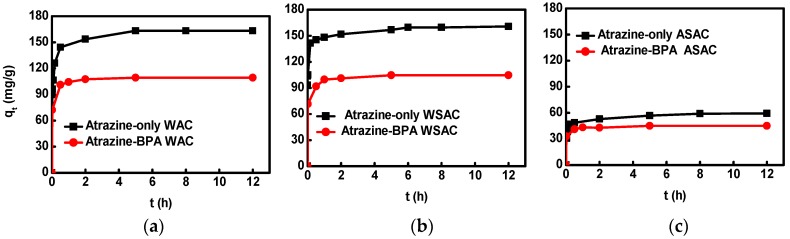
Influence of BPA on adsorption kinetics of atrazine onto WAC (**a**); WSAC (**b**); ASAC (**c**).

The order of the BET surface areas, total pore volume, and micropore volume of WSAC, WAC, and ASAC were WSAC > WAC > ASAC. In addition, the ASAC showed less porosity in the range of micropores and mesopores compared with WACand WSAC. According to another study, the high porosity of the adsorbent facilitated the diffusion of contaminants into the internal pore system of the adsorbent, and the high specific surface area may provide interfaces for the adsorption of contaminants [3]. Therefore, the adsorption capacity of WSAC and WAC was higher than that of ASAC. In addition, the large π bond may constitute between the π–π* of samples, and between the aromatic ring of BPA and atrazine. The adsorption behaviors of atrazine with/without BPA onto WAC, WSAC, and ASAC were similar. The adsorption rates of atrazine with/without BPA onto WAC, WSAC, and ASAC were the fastest at first 1 h, after that the rates slowed down, which was because the concentration of atrazine and the active sites of the adsorbent sample reduced as the reaction going on [22]. The adsorption progress reached the equilibrium during 5 h (Figure 2). The adsorption kinetic parameters of atrazine with/without BPA onto sample showed better accordance with the pseudo-second-order model rather than the pseudo-first-order model shown in Table 5, which implied the chemical adsorption mechanism contained in the adsorption process [20]. The *q*_e,cal_ from pseudo-second-order model was well consistent with the experimental data. The adsorption of atrazine on the ACs without BPA was affected by the porosity, specific surface area, and polarity of the ACs, as well as affected by π−π, H-bond, and hydrophobicity interactions [18]. Compared with the adsorption capacity of samples for atrazine in co-solution with BPA, the adsorption capacity of samples for atrazine without BPA was higher. According to the analysis about the adsorption kinetic of BPA in single solution, BPA could be absorbed by WAC, WSAC, and ASAC, and the adsorption performance of BPA was different in different samples. The hydrophobicity of BPA was stronger than atrazine, which lead to the hydrophobicity sites and pore structure of samples were occupied by BPA. In addition, the π–π interactions might occur between the aromatic ring of BPA and the aromatic compounds in samples, H-bonding interactions might occur between the hydrophilic groups (such as carboxyl and hydroxyl groups) of ACs and hydroxyl groups of BPA, and electrostatic attraction/repulsion might occur between BPA and ACs, which could lead to the adsorption active sites of atrazine occupied by BPA. 

### 3.2. Adsorption Isotherms

#### 3.2.1. Adsorption Isotherms of BPA onto ACs

**Figure 3:**
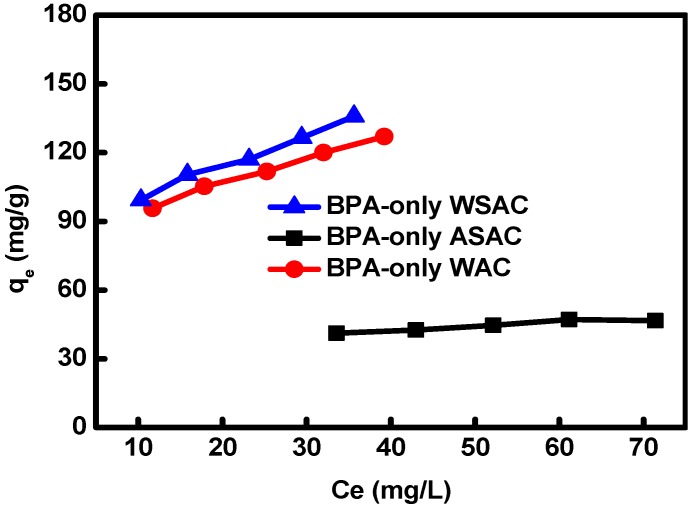
Adsorption isotherm of BPA onto ACs at 35 °C.

As illustrated in Figure 3, the adsorption isotherms showed that the adsorption capacity of BPA onto WAC and WSAC increased with the BPA concentration increasing. The increase of the initial BPA concentration reinforced the driving force and mass transfer between BPA and ACs [19]. There is a slight increase in the isotherm of BPA-only onto ASAC, due to the ASAC had a smaller specific surface area and more acidic functional groups. 

Compared with Freundlich, Temkin, and Dubinin-Radushkevic models, the Langmuir model was the best to describe the adsorption equilibrium of BPA onto WAC, WSAC, and ASAC (Table 6). It revealed that the adsorption process of BPA onto ACs was a molecular adsorption [20]. Based on the Langmuir model, according to *R*_L_, the adsorption process might be irreversible (*R*_L_ = 0), favorable (0 < *R*_L_ < 1), linear (*R*_L_ = 1), and unfavorable (*R*_L_ > 1). In this study, the value of *R*_L_ was in the range of 0–1, indicating that the adsorption of BPA onto samples was a favorable process [25]. This result was agreed with the reports by Qin, et al. and Libbrecht, et al. [26,27].

#### 3.2.2. Effect of BPA on Adsorption Isotherms of Atrazine on ACs 

**Figure 4:**
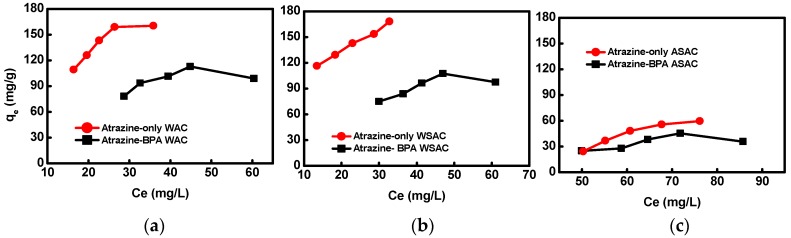
Effect of BPA on adsorption isotherms of atrazine onto WAC (**a**), WSAC (**b**), ASAC (**c**).

Adsorption isotherms showed that the adsorption capacity of atrazine onto WAC, WSAC, and ASAC increased with the atrazine concentration increasing. The adsorption capacity of ASAC, WAC, and WSAC for atrazine in the presence of BPA obviously decreased by 18.0%, 30.0%, and 30.3%, respectively (Figure 4), which indicated the competitive adsorption between BPA and atrazine. 

The porosity and specific surface area of WAC and WSAC were higher compared with those of ASAC, which was conducive to the adsorption for BPA. Therefore, the competitive adsorption between BPA and atrazine is more obvious onto WAC and WSAC, and then the adsorption capacity of atrazine in co-solution decreased more than that of ASAC [3]. In addition, the K_F_ value using WSAC in single solution is much higher than in the co-solution, and the K_F_ value increased and the 1/n decreased of WAC from the single to the co-solution, which was ascribed to the competitive adsorption in co-solution. The adsorption capacity of WSAC for atrazine in co-solution decreased more than that of WAC. The reason was that the porosity and specific surface area of WSAC were higher that WAC, thus, it was more favorable for the adsorption of BPA. The hydrophobicity of BPA was better than that of atrazine according to the value of log *K*_ow_ (Table 2) [28], leading to the different hydrophobic interactions with the adsorbents. According to the diameter and the structure of molecules, both of atrazine and BPA can enter the micropore structure of the adsorbents, resulting in the competitive adsorption between atrazine and BPA [3]. BPA might occupy the adsorption pores, thus reduce the adsorption capacity of adsorbents for atrazine. The competitive adsorption of BPA and atrazine might also be attributed to the interaction between BPA and the sample π−π, surface hydrophobic interaction, and hydrogen bonding, which reduced the effect of the adsorption of atrazine. Adsorption isotherms of ASAC, WAC, and WSAC for atrazine in co-solution were better fitted with the Freundlich model (Table 7), which was similar to that of single solute system.

### 3.3. Effect of HA on the Adsorption of Atrazine on ACs 

**Figure 5:**
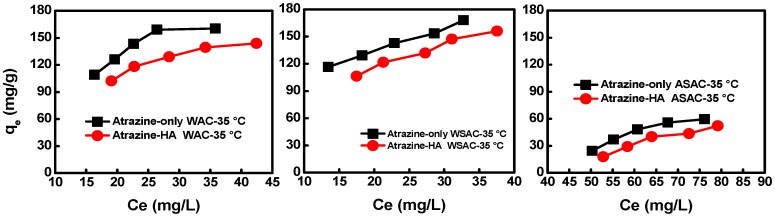
Effect of HA on the adsorption of atrazine onto WAC (**a**), WSAC (**b**), ASAC (**c**) (C_HA_ = 20 mg/L).

Based on the results of batch experiment, the adsorption capacity of samples for atrazine decreased significantly in the presence of HA, as shown in Figure 5, which was mainly because the large size of the HA molecule did not match with the small pore structure of ACs, resulting that HA could not be adsorbed into the pores [29]. However, the adsorption for HA were mainly due to the adsorbent electrostatic or hydrophobic interactions. The hydrophobicity of WAC was higher than that of WSAC and ASAC, and the adsorption capacity increased with the pore size of ACs increasing. Moreover, more mesopores of ACs could absorb more HA [30]. Therefore, HA might be adsorbed on the surface and occupy a small amount of active sites of samples. There was not much difference in the mesopores between WAC and WSAC, thereby the adsorption capacity of WAC and WSAC for atrazine in the presence of HA reduced almost same amount (Table 8). In addition, the R^2^ for the Freundlich fitting of atrazine in the presence of HA are comparable with all other models: WAC: 0.91, 0.98, 0.95 and 0.93, for WSAC: 0.90, 0.99, 0.98 and 0.91, ASAC: 0.95, 0.89 and 0.92, which indicated that the adsorption isotherm of atrazine in the presence of HA fitted well with the Freundlich model.

### 3.4. Effect of NaCl Concentrations on the Adsorption of Atrazine on ACs 

**Figure 6:**
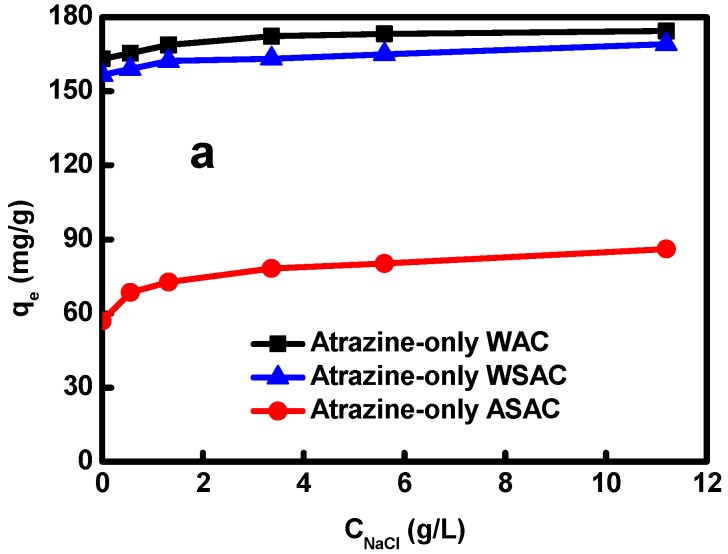
Effect of NaCl concentrations on the adsorption of atrazine on ACs.

The amount of atrazine adsorbed by the sample slightly increased in the presence of high NaCl concentration, and the increase of NaCl concentration had a little positive effect on the adsorption performance of atrazine onto ACs, as shown in Figure 6a. The possible reason was that the H-bond was formed between the water clusters and the H_2_O molecules, and then broke at the high concentration of NaCl, resulting in the appearing of the hydrophobic functional groups, which might improve the adsorption of atrazine by increasing amounts of NaCl [15]. 

### 3.5. Adsorption Mechanisms of Atrazine 

**Figure 7:**
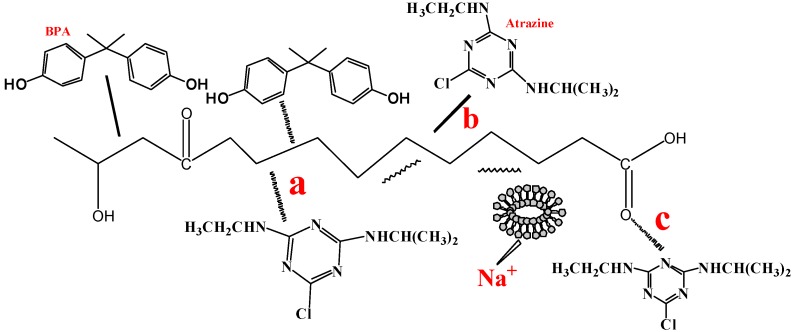
The adsorption mechanisms of atrazine for ACs onto samples in the presence of other substances (Notes: (**a**). π–π interactions; (**b**). Hydrophobic effect; (**c**). H-bond).

The presence of other substances is important for studying the effects of ASAC, WAC and WSAC on the adsorption mechanism for atrazine. According to our previous studies, the adsorption mechanisms of atrazine onto ACs in single solution mainly included the π–π interactions between the aromatic rings of samples and the heterocyclic ring of atrazine [31], the hydrophobic interactions between hydrophobic substances of samples and atrazine [3], and the H-bond between the carbonyl oxygen groups of the samples and N–H in the heterocycle of atrazine [32]. Raposo et al. also researched the adsorption mechanism of poly (o-methoxyaniline), and affirmed the chemical bonds by thermal stimulation adsorption experiments. The experimental results displayed that the adsorption process was controlled by the H-bond and electrostatic interactions [33]. When BPA coexisted with atrazine, the adsorption capacity of atrazine decreased obviously, which was because the value of *R*_L_ was within the range of 0–1, indicating that the adsorption of BPA onto sample was a favorable process. It mainly caused by the π–π interactions between the aromatic compounds in sample and the aromatic ring of BPA, the hydrophobic interactions between hydrophobic substances of samples and BPA, and the H-bonding interactions between oxygen-containing groups (such as carboxyl and hydroxyl groups) of ACs and hydroxyl groups of BPA [19], which lead to BPA occupied the adsorption site (Figure 7). In addition, the high micropore count of samples promoted the adsorption capacity for atrazine and BPA, which lead to BPA occupied the active sites of atrazine. When HA or NaCl coexisted with atrazine, the adsorption capacity of atrazine onto samples slightly decreased. When HA coexisted with atrazine, the competitive adsorption appeared, which was mainly caused by HA absorbed onto the adsorbent surface [26]. The adsorption mechanisms for HA were mainly the electrostatic or hydrophobic interactions: HA occupied a small amount of active sites; the coexisting cations in tap water occupied the adsorption site of atrazine [15]. When the high concentration of NaCl coexisted with atrazine in solution, the adsorption capacity of atrazine increased slightly, which is caused by the expositing of the sample’s hydrophobic functional groups: H-bonds, forming between the water clusters and the H_2_O molecules on the sample surface, was broken at the high concentration of NaCl, which was beneficial to improve the adsorption capacity of atrazine [15].

## 4. Conclusions

Adsorption kinetics of ASAC, WAC, and WSAC for atrazine was fitted with the pseudo-second-order kinetics model, and the isotherm was consistent with the Freundlich model with/without BPA in solution. When BPA coexisted with atrazine in solution, the adsorbed atrazine amount onto samples decreased. The competitive adsorption between atrazine and BPA onto samples might be attributed to the π−π interactions, hydrophobic interactions, and H-bonds, which lead to BPA occupied the active site of activated carbon for atrazine. In addition, in the co-solution of HA and atrazine, the adsorbed atrazine amount decreased significantly because the active sites for atrazine were occupied by the HA. Moreover, an increased concentration of NaCl in the co-solution was beneficial to the adsorption of atrazine by the samples due to the increased broken H-bonds, which leads to the expositing of hydrophobic functional groups of the samples. 

## Figures and Tables

**Table 1:** Elemental composition and textural characteristics of the adsorbents.

Parameters	ASAC	WAC	WSAC
N%	0.31	0.30	0.58
C%	47.98	72.86	67.88
H%	0.58	0.52	0.89
S%	0.66	1.32	0.91
O,diff%	50.47	25.00	29.74
N/C atomic ratio	0.006	0.004	0.009
H/Catomic ratio	0.012	0.007	0.013
O/C atomic ratio	1.052	0.341	0.438
(O+N)/C atomic ratio	1.058	0.345	0.447
BET specifics surface area (m^2^/g)	276.15	553.33	614.21
Total pore volume (cm^3^/g)	0.21	0.41	0.46
Micropore volume (cm³/g)	0.12	0.25	0.28
Average pore size (nm)	3.69	3.41	3.40
π-π*	4.76×10^−5^	5.24	7.67

**Table 2:** Major physicochemical properties of atrazine and BPA.

Chemicals	Structural Formula	log K_ow_	Molecular Size (nm^3^)	References
Atrazine	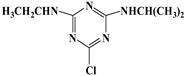	2.18	0.96×0.84×0.3	[12]
BPA	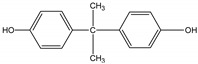	3.32	0.94×0.53×0.43	[20]

(Note: log K_ow_ = Octanol-water partition coefficient).

**Table 3:** The pH_pzc_and Boehm titration results of the WAC, WSAC, and ASAC samples

Samples	pH_pzc_	Carboxyl mmol/g	Lactone mmol/g	Phenol mmol/g	Total acidic mmol/g	Total basic mmol/g
*WAC*	7.11	0.29	0.31	0.31	0.91	0.21
*WSAC*	7.26	0.21	0.29	0.26	0.76	0.26
*ASAC*	6.83	0.41	0.36	0.37	1.14	0.15

**Table 4:** Adsorption kinetic parameters of BPA onto ACs in single solution.

Adsorbents	q_e,exp_ (mg/g)	Pseudo-First-Order				Pseudo-Second-Order			
		q_e,cal_ (mg/g)	k _l_ (h^−1^)	R^2^	RSEM	q_e, cal_ (mg/g)	k_2_(g/(mg∙h)	R^2^	RSEM
*WAC*	131.01	18.87	1.20	0.32	110.31	131.58	0.47	0.99	3.31
*WSAC*	136.26	29.48	1.89	0.62	98.87	136.98	0.53	0.99	3.42
*ASAC*	51.91	19.00	1.37	0.89	32.19	52.91	0.21	0.99	2.98

**Table 5:** Influence of BPA on adsorption kinetic parameters of atrazine onto ACs.

Adsorbents	Adsorbate	q_e,exp_ (mg/g)	Pseudo-First-Order				Pseudo-Second-Order			
			q_e,cal_ (mg/g)	k _l_ (h^−^^1^)	R^2^	RSEM	q_e, cal_ (mg/g)	k_2_(g/(mg∙h)	R^2^	RSEM
WAC	Atrazine	163.09	21.70	0.08	0.05	138.87	158.73	0.39	0.99	4.79
WSAC	Atrazine	156.62	29.48	0.43	0.43	124.65	156.25	0.51	0.99	4.38
ASAC	Atrazine	56.91	6.22	0.27	0.14	45.76	57.18	1.02	0.99	3.90
WAC	Atrazine with BPA	109.44	26.32	1.33	0.81	76.45	109.89	0.21	0.99	2.99
WSAC	Atrazine with BPA	104.48	29.50	1.12	0.01	68.54	105.26	0.18	0.99	3.03
ASAC	Atrazine with BPA	45.14	5.86	0.11	0.90	35.21	45.05	0.20	0.99	2.76

**Table 6:** Adsorption isotherm parameters of BPA onto ACs.

Adsorption Isotherm Models	Parameters	WAC	WSAC	ASAC
Langmuir	q_m_(mg/g)	138.89	156.25	57.14
	K_L_(L/mg)	0.23	0.15	0.09
	R^2^	0.99	0.99	0.99
	RSEM	3.16	2.54	2.16
Freundlich	K_F_ (L/mg)	54.15	57.69	21.22
	1/n	0.22	0.24	0.19
	R^2^	0.87	0.88	0.86
	RSEM	3.17	4.36	3.36
Temkin	A (L/mg)	0.277	0.32	8.27
	B	25.31	28.09	0.24
	R^2^	0.88	0.76	0.72
	RSEM	54.03	60.43	23.04
Dubinin-Radushkevic	q_m_(mg/g)	138.61	147.35	53.04
	K_d_	4.72	4.47	9.12
	R^2^	0.89	0.83	0.70
	RSEM	4.53	3.93	4.83

**Table 7:** Effect of BPA on adsorption isotherm parameters of atrazine onto ACs.

Adsorption Isotherm Models	Parameters	Atrazine in Single Solution			Atrazine in co-Solution with BPA		
		WAC	WSAC	ASAC	WAC	WSAC	ASAC
Langmuir	q_m_(mg/g)	303.00	294.12	46.30	125	142.85	45.25
	K_L_(L/mg)	0.04	0.037	0.007	0.085	0.043	0.007
	R^2^	0.71	0.90	0.45	0.85	0.82	0.68
	RSEM	11.47	10.00	32.60	9.53	7.77	23.83
Freundlich	K_F_ (L/mg)	5.13	25.58	1.10	6.26	4.68	0.028
	1/n	1.06	0.53	0.924	0.76	0.81	1.72
	R^2^	0.92	0.99	0.89	0.95	0.98	0.92
	RSEM	6.42	2.81	1.36	3.17	1.75	2.33
Temkin	A (L/mg)	56.82	71.27	71.30	9.35	134.29	0.029
	B	0.42	0.29	0.029	72.15	0.017	58.08
	R^2^	0.73	0.89	0.81	0.96	0.62	0.90
	RSEM	10.51	11.16	5.73	23.11	13.63	9.59
Dubinin-Radushkevic	q_m_(mg/g)	221.78	228.47	255.98	208.89	145.37	179.00
	K_d_	12.73	12.26	116.22	28.01	102.34	19.15
	R^2^	0.82	0.87	0.81	0.95	0.72	0.89
	RSEM	9.54	10.24	5.92	19.18	7.43	9.78

**Table 8:** Effect of HA on adsorption isotherm parameters of atrazine onto ACs.

Adsorption Isotherm Models	Parameters	Atrazine in Single Solution			Atrazine in Co-solution with HA		
		WAC	WSAC	ASAC	WAC	WSAC	ASAC
Langmuir	q_m_ (mg/g)	303.00	294.12	46.30	212.77	263.16	NA
	K_L_ (L/mg)	0.04	0.037	0.007	0.65	0.0038	NA
	R^2^	0.71	0.90	0.45	0.91	0.90	NA
Freundlich	K_F_ (L/mg)	5.13	25.58	1.10	30.61	25.05	0.0012
	1/n	1.06	0.53	0.924	0.42	0.50	2.47
	R^2^	0.92	0.99	0.89	0.98	0.99	0.95
Temkin	A (L/mg)	56.82	71.27	71.30	0.40	0.27	0.024
	B	0.42	0.29	0.029	52.42	63.03	79.93
	R^2^	0.73	0.89	0.81	0.95	0.98	0.89
Dubinin-Radushkevic	q_m_ (mg/g)	221.78	228.47	255.98	197.06	220.03	433.89
	K_d_	12.73	12.26	116.22	12.37	13.19	164.78
	R^2^	0.82	0.87	0.81	0.93	0.91	0.92
